# Expression of Foxm1 Transcription Factor in Cardiomyocytes Is Required for Myocardial Development

**DOI:** 10.1371/journal.pone.0022217

**Published:** 2011-07-14

**Authors:** Craig Bolte, Yufang Zhang, I-Ching Wang, Tanya V. Kalin, Jeffrey D. Molkentin, Vladimir V. Kalinichenko

**Affiliations:** 1 Division of Pulmonary Biology, Cincinnati Children's Hospital Research Foundation, Cincinnati, Ohio, United States of America; 2 Division Molecular Cardiovascular Biology, Cincinnati Children's Hospital Research Foundation, Cincinnati, Ohio, USA; University of Texas M. D. Anderson Cancer Center, United States of America

## Abstract

Forkhead Box M1 (Foxm1) is a transcription factor essential for organ morphogenesis and development of various cancers. Although complete deletion of Foxm1 in *Foxm1^−/−^* mice caused embryonic lethality due to severe abnormalities in multiple organ systems, requirements for Foxm1 in cardiomyocytes remain to be determined. This study was designed to elucidate the cardiomyocyte-autonomous role of Foxm1 signaling in heart development. We generated a new mouse model in which Foxm1 was specifically deleted from cardiomyocytes (*Nkx2.5-Cre/Foxm1^fl/f^* mice). Deletion of Foxm1 from cardiomyocytes was sufficient to disrupt heart morphogenesis and induce embryonic lethality in late gestation. *Nkx2.5-Cre/Foxm1^fl/fl^* hearts were dilated with thinning of the ventricular walls and interventricular septum, as well as disorganization of the myocardium which culminated in cardiac fibrosis and decreased capillary density. Cardiomyocyte proliferation was diminished in *Nkx2.5-Cre/Foxm1^fl/fl^* hearts owing to altered expression of multiple cell cycle regulatory genes, such as Cdc25B, Cyclin B_1_, Plk-1, nMyc and p21^cip1^. In addition, Foxm1 deficient hearts displayed reduced expression of CaMKIIδ, Hey2 and myocardin, which are critical mediators of cardiac function and myocardial growth. Our results indicate that Foxm1 expression in cardiomyocytes is critical for proper heart development and required for cardiomyocyte proliferation and myocardial growth.

## Introduction

The heart is the first organ to function during embryonic development, the beating heart can be detected as early as embryonic day 8 (E8) in the mouse [1], [2]. Proper cardiac development requires strict adherence to a temporal and spatial pattern of gene expression. Embryonic development of the heart is mediated by proliferative growth, with cardiomyocytes rapidly progressing through the cell cycle and multiplying [3]. In the postnatal period, cardiomyocytes withdrawal from the cell cycle and cardiac growth becomes dependent on hypertrophy of individual cardiomyocytes [3]. Transcriptional regulation of cardiomyocyte proliferation during embryogenesis has been extensively studied, and several cardiac transcription factors were found to be critical for cardiomyocyte progression into the cell cycle. These include GATA family members 4 and 6 [4], myocardin [5], Twist family members 1 [6] and 2 [7], Hey2 [8], [9], Sox4 [10] and Nkx2.5 [11].

Foxm1 (previously known as HFH-11B, Trident, Win, or MPP2) is a member of the Forkhead Box (Fox) family of transcription factors which share homology in the Winged Helix/Forkhead DNA binding domain. Foxm1 is expressed in proliferating cells of all embryonic tissues, including cardiac progenitor cells and the early myocardium [12], [13]. However, expression wanes postnatally and Foxm1 can only be detected in a few adult tissues such as intestinal crypts, thymus and testis [14], [15]. Foxm1 signaling has been shown to be a critical mediator of both G_1_-S and G_2_-M transitions of the cell cycle, and to be upregulated in various human cancers [16], [17], [18], [19], [20], [21]. In addition, Foxm1 was determined to play a role in tissue repair following injury in the lungs and liver [15], [22], [23].

Foxm1-null (*Foxm1^−/−^*) mouse lines have been previously generated and characterized by two separate labs [24], [25]. *Foxm1^−/−^* mice in which the DNA binding and C-terminal transcriptional activation domains of the Foxm1 protein were deleted die *in utero* between E13.5 and E16.5 due to multiple abnormalities in various organ systems, including liver, lungs, blood vessels, brain and heart [13], [25], [26], [27]. Although these studies showed that Foxm1 plays a cell autonomous role for organ development in multiple cell types, the role of Foxm1 in cardiac development and function remains unknown. Given widespread organ defects in *Foxm1^−/−^* mice, it remains unclear whether Foxm1 is critical for heart development or if cardiac abnormalities are secondary to defects in other organ systems which could alter embryonic growth. Therefore, a direct role of Foxm1 in cardiomyocyte growth and/or function awaits elucidation.

As Foxm1 is widely expressed during embryogenesis [12], [28], [29], the recent focus has been to elucidate the cell-specific roles of Foxm1 in different tissues using conditional knockout mouse models. Specific deletion of Foxm1 from hepatoblasts resulted in embryonic lethality around day E18.5 with disruption of hepatic cords and vasculature, as well as a lack of intrahepatic bile ducts [25]. Deletion of Foxm1 from precursors of cerebellar granule neurons interfered with Shh-induced signaling to delay brain development [30]. Foxm1 deletion from T lymphocyte lineage decreased proliferation of early thymocytes and activated mature T cells without affecting apoptosis or T cell differentiation [31]. However, mice with endothelial- or macrophage-specific Foxm1 deletions developed normally [32], [33], indicating Foxm1 is dispensable in these cells lines during embryogenesis. Furthermore, while deletion of Foxm1 specifically from the pancreas did not affect pancreatic development [34], male mice developed islet dysfunction and diabetes resulting from impaired postnatal β-cell mass expansion [34] and females were prone to gestational diabetes [35], indicating Foxm1 requirements differ during pancreatic development. Deletion of Foxm1 specifically from smooth muscle cells did not affect differentiation, but mice died immediately after birth from severe pulmonary hemorrhage, structural defects in the arterial wall and esophageal abnormalities [28]. When Foxm1 was deleted conditionally in developing respiratory epithelium proliferation rates of respiratory epithelial cells were unaltered [12], suggesting that Foxm1 is not required for epithelial proliferation during lung development. However, deletion of Foxm1 from respiratory epithelium impaired lung maturation, decreased expression of surfactant-associated proteins SPA, SPB and SPC and delayed differentiation of type I cells from epithelial precursors causing respiratory failure after birth [12]. Thus, Foxm1 is essential for surfactant homeostasis and lung maturation during lung development. Studies in conditional Foxm1 knockout models have shown that Foxm1 plays unique roles in different tissues during embryonic development; the cardiomyocyte-specific role of Foxm1 in heart development remains unexplored.

In this study, we utilized the Cre-LoxP system to conditionally delete Foxm1 from cardiomyocytes to ascertain the cardiomyocyte-autonomous role of Foxm1 in heart development. Deletion of Foxm1 from cardiomyocytes resulted in chamber dilation and myocardial thinning, culminating in embryonic lethality in late gestation. Cardiac Foxm1 deletion caused decreased cardiomyocyte proliferation and altered expression of cell cycle regulators Cdc25B, Cyclin B_1_, nMyc, Plk-1 and p21^cip1^. We also identified CaMKIIδ, Hey2 and myocardin as new potential targets of Foxm1 signaling and mediators of myocardial thinning. This study shows that Foxm1 is critical for expression of cell cycle regulatory genes in developing cardiomyocytes and is required for proper heart development.

## Methods

### Foxm1 conditional knockout mice

We have previously described the generation of *Foxm1* LoxP/LoxP (*Foxm1^fl/fl^*) mice, in which LoxP sequences flank exons 4 through 7 of the *Foxm1* gene encoding the DNA binding and transcriptional activation domains [25]. *Foxm1^fl/fl^* mice were bred with *Nkx2.5-Cre* mice [36] to generate *Nkx2.5-Cre/Foxm1^fl/fl^* double transgenic mice with deletion of Foxm1 from the myocardium. Using lineage tracing experiments previous studies demonstrated that *Nkx2.5-Cre* was expressed in the early myocardium as well as epithelium of the first pharyngeal arch [36]. However, no gross morphological abnormalities were observed in any non-cardiac tissues examined including thyroid and thymus (data not shown). *Nkx2.5-Cre/Foxm1^fl/fl^* homozygous embryos exhibited an embryonic lethal phenotype with the exception of one mouse from an early breeding which survived to postnatal day 11 (P11). To generate *Nkx2.5-Cre/Foxm1^fl/fl^* embryos, *Nkx2.5-Cre/Foxm1^fl/+^* heterozygous males were bred with *Foxm1^fl/fl^* females. *Foxm1^fl/fl^* or *Nkx2.5-Cre/Foxm1^fl/+^* embryos from the same litter were used as controls. Animal studies were reviewed and approved by the Animal Care and Use Committee of Cincinnati Children's Hospital Research Foundation.

### Immunohistochemical staining

*Nkx2.5-Cre/Foxm1^fl/fl^* and control embryos were harvested on embryonic day 14.5 (E14.5) and E17.5. Embryos were then fixed in 4% paraformaldehyde overnight and embedded into paraffin blocks. Paraffin sections of 5 µm were used for immunohistocheshow

### Quantitative real-time RT-PCR (qRT-PCR)

Total cardiac RNA was prepared from individual *Nkx2.5-Cre/Foxm1^fl/fl^* and control hearts using RNA-STAT-60 (Tel-Test “B” Inc. Friendswood, TX). cDNA was generated using the Applied Biosystems High Capacity cDNA Reverse Transcription kit (Applied Biosystems, Foster City, CA). Evaluation of expression levels of specific genes was performed by qRT-PCR using Taqman probes (Table 1) and StepOnePlus Real-Time PCR system (Applied Biosystems, Foster City, CA) as previously described [12], [20], [28].

**Table 1:** TaqMan gene expression assays (Applied Biosystems) used for qRT-PCR analysis.

Mouse Foxm1	Mm00514924_m1
Mouse Cdc25B	Mm00499136_m1
Mouse Cyclin B_1_	Mm00838401_g1
Mouse Cyclin D_1_	Mm00432359_m1
Mouse p21^cip1^	Mm01303209_m1
Mouse Plk-1	Mm00440924_g1
Mouse cMyc	Mm00487804_m1
Mouse nMyc	Mm00476449_m1
Mouse CaMKIIδ	Mm00499266_m1
Mouse β-catenin	Mm00437992_m1
Mouse NFATc3	Mm01249200_m1
Mouse GATA4	Mm00484689_m1
Mouse GATA6	Mm00802636_m1
Mouse Hey2	Mm00469280_m1
Mouse Myocardin	Mm00455051_m1
Mouse Twist1	Mm00442036_m1
Mouse Twist2	Mm00492147_m1
Mouse Sox4	Mm00486320_s1
Mouse Notch1	Mm00435245_m1
Mouse Notch2	Mm00440536_g1
Mouse FGF10	Mm00433275_m1
Mouse IL-1β	Mm01336189_m1
Mouse TGF-β_1_	Mm03024053_m1
Mouse BMP2	Mm01340178_m1
Mouse BMP4	Mm01321704_m1
Mouse Wnt5a	Mm00437347_m1
Mouse Wnt7b	Mm00437357_m1
Mouse β-actin	Mm00607939_s1

### Western blot analysis

Hearts from E14.5 embryos were harvested and used to prepare protein extract. Three hearts from embryos with matching genotypes were pooled. Protein extracts were run on PAGE-SDS gels and transferred to PVDF membranes followed by incubation with primary antibodies specific for Foxm1 (1∶1000; C20; Santa Cruz), Cyclin B_1_ (1∶500; BD Pharmingen), or p21^cip1^ (1∶300; BD Pharmingen). Secondary antibodies were conjugated with horse-radish peroxidase. β-actin was used as a loading control.

### Statistical analysis

Student's T-test was used to determine statistical significance. P values <0.05 were considered significant. Values for all measurements were expressed as mean±standard error of mean (SEM).

## Results

### Foxm1 protein and mRNA expression declines during embryonic and postnatal development

**Figure 1:**
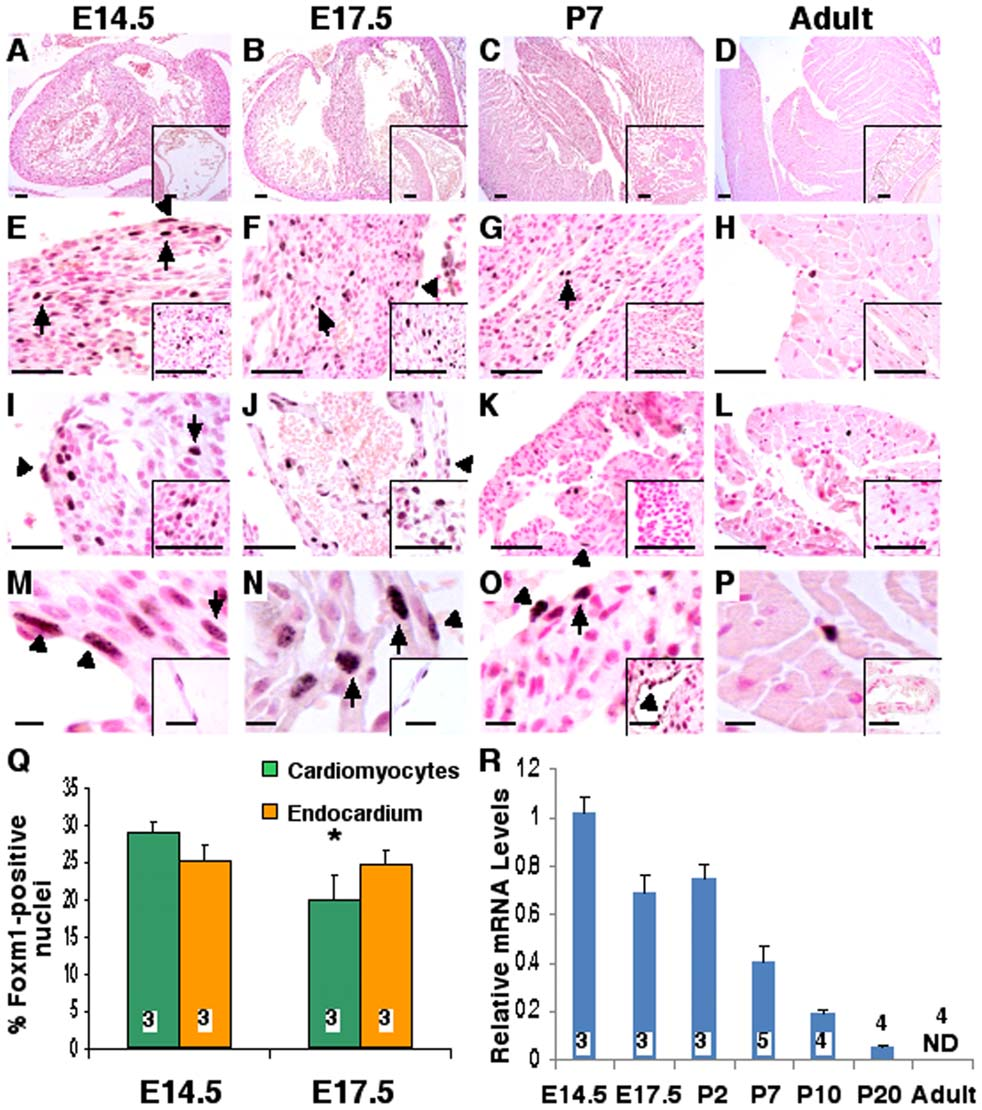
Foxm1 expression during heart development. Foxm1 protein was evaluated in microtome sections of paraffin-embedded hearts from embryonic day 14.5 (E14.5) (A, E, I, M), E17.5 (B, F, J, N), postnatal day 7 (P7) (C, G, K, O), and adult wild type mice (D, H, L, P) by immunohistochemistry with Foxm1 antibodies [37] and counterstained with nuclear fast red (red nuclei). Foxm1 was detected in cardiomyocytes (arrows) and endocardial cells (arrowheads) from the ventricles (E–H, M–P), interventricular septum (inset E–H), and atrial walls (I–L) throughout cardiac development although expression progressively waned. Foxm1 was also detected in valves at E14.5 and E17.5 (inset I–J) but not postnatally (inset K–L). Foxm1-positive nuclei were detected in the embryonic pericardium (inset M–N) and in the coronary vasculature (inset O–P) until P7. The number of Foxm1-positive nuclei decreased during gestation in cardiomyocytes but was unaltered in endocardial cells (Q). Mean±SEM was determined from 5 random sections at E14.5 and P7 with 3 hearts in each group. qRT-PCR of total heart RNA demonstrated a decrease in Foxm1 mRNA from E14.5 to P20 and Foxm1 mRNA was undetectable in the adult heart (R). Foxm1 expression was normalized to β-actin mRNA. Significant differences (p<0.05) were indicated by asterisk. “N” values were represented by boxes inside or above bars. Scale bars represent 100 µm in A–D insets, 200 µm in A–D, 50 µm in E–L (main and insets) and 10 µm in M–P (main and insets).

Hearts were collected from wild type mice at multiple timepoints during cardiac development. To identify cells expressing Foxm1, microtome sections of paraffin-embedded hearts were immunohistochemically stained using anti-Foxm1 antibodies. Foxm1 protein was highly abundant in the embryonic period and easily detectable in nuclei of cardiomyocytes and endothelial cells up to postnatal day 7 (P7) (Figure 1A–H). Foxm1 staining was observed throughout the developing heart, in the ventricles (Figure 1E–H and M–P), interventricular septum (insets of Figure 1E–H), atria (Figure 1I–L), heart valves (insets of Figure 1I–L), pericardium (insets of Figure 1M–N) and vasculature (insets of Figure 1O–P). The percentage of Foxm1-positive cardiomyocytes decreased from 29% at E14.5 to 20% at P7 (Figure 1Q) and nuclear Foxm1 staining was seldom observed in the adult heart as only 4±1% of cardiomyocytes were Foxm1-positive (data not shown). In addition, hearts were used to isolate total RNA and Foxm1 mRNA expression was examined by qRT-PCR. Similar to Foxm1 protein expression, Foxm1 mRNA was most highly expressed in the early embryonic period. Foxm1 expression declined by 30% from E14.5 to E17.5, but maintained a similar level of expression until P2. Foxm1 mRNA levels decreased rapidly in the postnatal period from P2 to P20, and Foxm1 mRNA was undetectable in the adult heart (11 weeks) (Figure 1R).

### Cardiomyocyte-specific deletion of Foxm1 causes embryonic lethality

**Figure 2:**
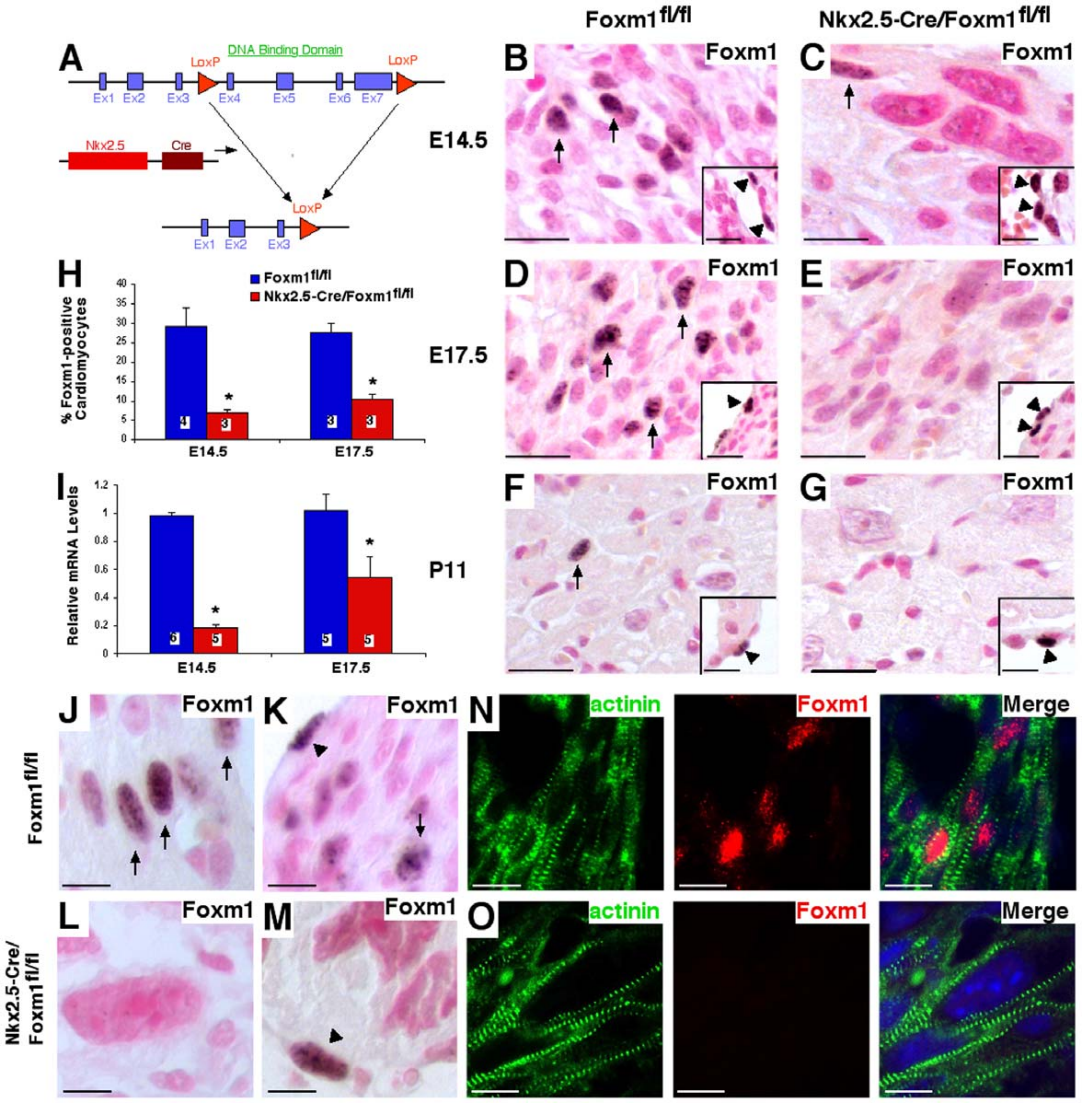
Generation of *Nkx2.5-Cre/Foxm1^fl/fl^* mice and efficiency of Foxm1 deletion. The Cre-LoxP system was utilized to generate a mouse line in which Foxm1 was selectively deleted from myocardial cells (A). *Foxm1^fl/fl^* mice were crossed with mice expressing *Nkx2.5-Cre* to generate a mouse line in which Foxm1 is truncated in cardiomyocytes early in embryonic development. Microtome sections of paraffin-embedded hearts were prepared from *Nkx2.5-Cre/Foxm1^fl/fl^* and control (*Foxm1^fl/fl^*) mice at E14.5 (B–C, J–O), E17.5 (D–E) and P11 (F–G) and stained with Foxm1 antibodies. Foxm1 was observed in myocardial (arrows) and endocardial (arrowheads) cells. Quantification of the percentage of Foxm1-positive nuclei showed that cardiomyocytes positive for Foxm1 were significantly decreased in *Nkx2.5-Cre/Foxm1^fl/fl^* hearts compared to control at E14.5 and E17.5 (H). Similarly, Foxm1 mRNA was decreased in *Nkx2.5-Cre/Foxm1^fl/fl^* hearts at E14.5 and E17.5 as determined by qRT-PCR (I). Foxm1 expression was normalized to β-actin mRNA. Foxm1 was more readily detectable in cardiomyocytes from control (J–K, N) than *Nkx2.5-Cre/Foxm1^fl/fl^* hearts (L–M, O) as evidenced by colocalization of Foxm1-positive nuclei with α-actinin (N–O). However, Foxm1-positive endocardial cells could be detected in control (K) and *Nkx2.5-Cre/Foxm1^fl/fl^* hearts (M). Significant differences (p<0.05) were indicated by asterisk. “N” values were represented by boxes inside bars. Scale bars represent 200 µm in B–G and 50 µm in J–O.

Previous studies demonstrated that complete deletion of Foxm1 (*Foxm1^−/−^* mice) caused embryonic lethality between E13.5 and E16.5 and presented with malformation of multiple organ systems [25]. To ascertain the cardiomyocyte-autonomous role of Foxm1 signaling in cardiac development, *Foxm1^fl/fl^* mice (in which LoxP sites flank exons 4–7) were crossed with *Nkx2.5-Cre* mice to generate a mouse line in which the DNA binding and transcriptional activation domains of the Foxm1 protein were excised in cardiomyocytes (Figure 2A). Breeding pairs between *Foxm1^fl/fl^* and *Nkx2.5-Cre/Foxm1^fl/+^* heterozygous mice were used to generate embryos with the *Nkx2.5-Cre* transgene and homozygous for the *Foxm1^fl/fl^* allele (*Nkx2.5-Cre/Foxm1^fl/fl^*) at an expected ratio of 1∶4. The majority of *Nkx2.5-Cre/Foxm1^fl/fl^* embryos did not survive to birth (Table 2). Although a near Mendelian ratio was observed for *Nkx2.5-Cre/Foxm1^fl/fl^* embryos at E14.5 (Table 2), between E14.5 and E17.5 nearly 50% of *Nkx2.5-Cre/Foxm1^fl/fl^* embryos were lost, with the remaining 50% lost between E17.5 and birth (Table 2). Only one *Nkx2.5-Cre/Foxm1^fl/fl^* pup survived to postnatal day 11 but exhibited severe growth retardation prior to harvest (data not shown). Thus, Foxm1 deletion from cardiomyocytes is sufficient to induce embryonic lethality.

**Table 2:** Breeding table Nkx2.5-Cre/Foxm1^fl/+^ X Foxm1^fl/fl^.

	E14.5	E17.5	Newborns
Total embryos/pups	31	46	67
Expected ratio of *Nkx2.5-Cre/Foxm1^fl/fl^*	0.25	0.25	0.25
Experimental ratio of *Nkx2.5-Cre/Foxm1^fl/fl^*	0.23	0.13	0.01
% lethality of *Nkx2.5-Cre/Foxm1^fl/fl^*	9.68	47.83	94.03

Cumulative breeding data between *Nkx2.5-Cre/Foxm1^fl/+^* and *Foxm1^fl/fl^* mice shows significant lethality in *Nkx2.5-Cre/Foxm1^fl/fl^* embryos by day 17.5 (E17.5). 47% of *Nkx2.5-Cre/Foxm1^fl/fl^* embryos died by E17.5 and 94% lethality was observed in *Nkx2.5-Cre/Foxm1^fl/fl^* embryos at birth.

Efficiency of Foxm1 deletion from cardiomyocytes and endocardial cells was determined by immunohistochemical staining with antibodies against Foxm1. The percentage of Foxm1-positive cardiomyocyte nuclei was significantly decreased in *Nkx2.5-Cre/Foxm1^fl/fl^* embryos compared to littermate controls (Figure 2H). However, the percentage of Foxm1-positive endocardial cells was unchanged (41.2±2.7% vs. 40.9±5.4% at E14.5 and 37.1±1.4% vs. 38.8±1.3% at E17.5 for *Foxm1^fl/fl^* and *Nkx2.5-Cre/Foxm1^fl/fl^* respectively, Figure 2K and M, insets B–G), indicating specificity of Foxm1 deletion to cardiomyocytes. Cardiomyocyte Foxm1 staining was decreased by as much as 77% at E14.5 and was still decreased by nearly 63% at E17.5 (Figure 2H). The observed efficiency of Foxm1 deletion in *Nkx2.5-Cre/Foxm1^fl/fl^* hearts was substantiated by qRT-PCR analysis of total heart RNA. Compared to *Foxm1^fl/fl^* littermates, *Foxm1* mRNA was decreased by 82% and 47% in *Nkx2.5-Cre/Foxm1^fl/fl^* hearts at E14.5 and E17.5, respectively (Figure 2I).

### Embryonic deletion of Foxm1 from cardiomyocytes causes myocardial thinning, ventricular hypoplasia and disorganization of the myocardium

**Figure 3:**
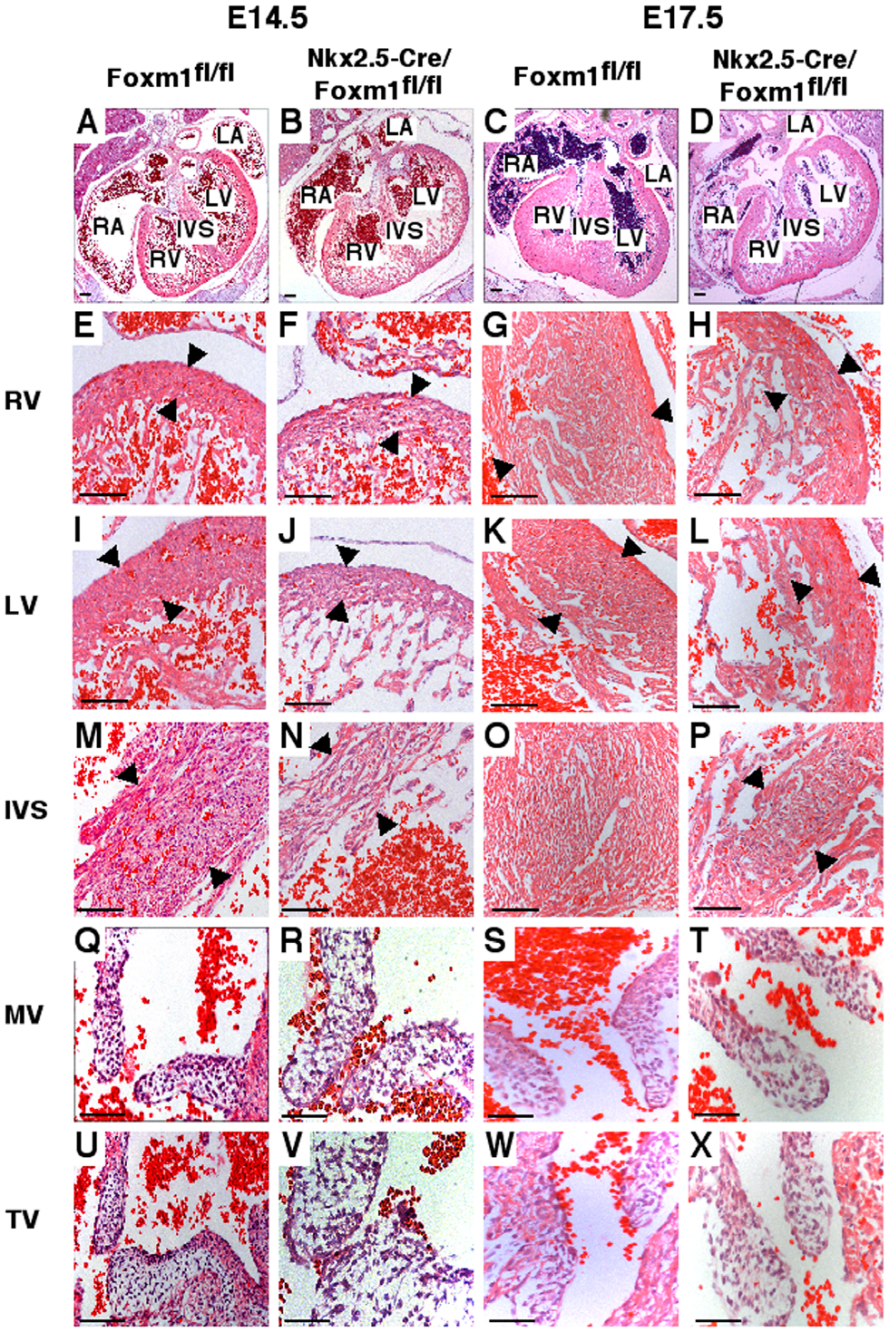
Myocardial thinning and cardiomyocyte disarray in *Nkx2.5-Cre/Foxm1^fl/fl^* mice. Microtome sections of paraffin-embedded hearts were prepared from *Nkx2.5-Cre/Foxm1^fl/fl^* and control *Foxm1^fl/fl^* mice at E14.5 and E17.5 and stained with hematoxylin and eosin (H&E). H&E staining showed that hearts from *Nkx2.5-Cre/Foxm1^fl/fl^* embryos possessed all four chambers and were similar in size to littermate control hearts (A–D). However, there was thinning of the muscular wall of the right ventricle (RV) (E–H), left ventricle (LV) (I–L), and interventricular septum (IVS) (M–P) in addition to disorganization of the musculature. There was no difference in the size of the leaflets in either the mitral (MV) (Q–T) or tricuspid valves (TV) (U–X). Arrowheads indicate area where measurements of thickness were made. Scale bars represent 100 µm in A–B and E–P, 200 µm in C–D and 50 µm in Q–X.

Hearts from *Nkx2.5-Cre/Foxm1^fl/fl^* embryos exhibited abnormal cardiac morphology in comparison to *Foxm1^fl/fl^* and *Nkx2.5-Cre/Foxm1^fl/+^* littermate controls. The ventricular lumen was dilated and ventricular walls were thinner in Foxm1 deficient embryos compared to control embryos (Figure 3A–L). Ventricular wall thickness was significantly decreased at E14.5 and E17.5 (Figure 3E–L, Table 3). Thickness of the interventricular septum (IVS) was decreased by 42% and 62% at E14.5 and E17.5, respectively, (Figure 3M–P, Table 3) and cardiomyocyte organization within the IVS was in disarray (Figure 3M–P). There was, however, no significant change in the overall size of the heart at these two embryonic timepoints (Figure 3A–D). Furthermore, although an increased network of extracellular matrix was observed in valves from *Nkx2.5-Cre/Foxm1^fl/fl^* mice, there was no overall effect on valve size by deletion of Foxm1 from cardiomyocytes (Figure 3Q–X). In addition, there were no gross morphological changes in other organs known to delineate from Nkx2.5 expressing cells such as the thymus (data not shown).

**Table 3:** Cardiac morphologic parameters.

	E14.5			E17.5		
	RV (µm)	LV (µm)	IVS (µm)	RV (µm)	LV (µm)	IVS (µm)
*Foxm1^fl/fl^*	75.3±5.0	104.2±9.7	222.9±23.8	172.3±5.2	143.4±5.5	369.5±20.0
*Nkx2.5- Cre/Foxm1^fl/fl^*	63.0±8.4	59.6±5.1*	142.2±24.2*	98.5±4.4*	94.6±7.4*	159.1±12.6*

Hearts from *Nkx2.5-Cre/Foxm1^fl/fl^* embryos had significantly decreased myocardial thickness compared to littermate control mice at E14.5 and E17.5. Five sections from 3 *Foxm1^fl/fl^* and *Nkx2.5-Cre/Foxm1^fl/fl^* embryos were measured to calculate mean±SEM. Significant differences (p<0.05) shown with asterisks. Abbreviations: RV, right ventricle; LV, left ventricle; IVS, interventricular septum.

### Decreased cardiomyocyte proliferation in *Nkx2.5-Cre/Foxm1^fl/fl^* embryos

**Figure 4:**
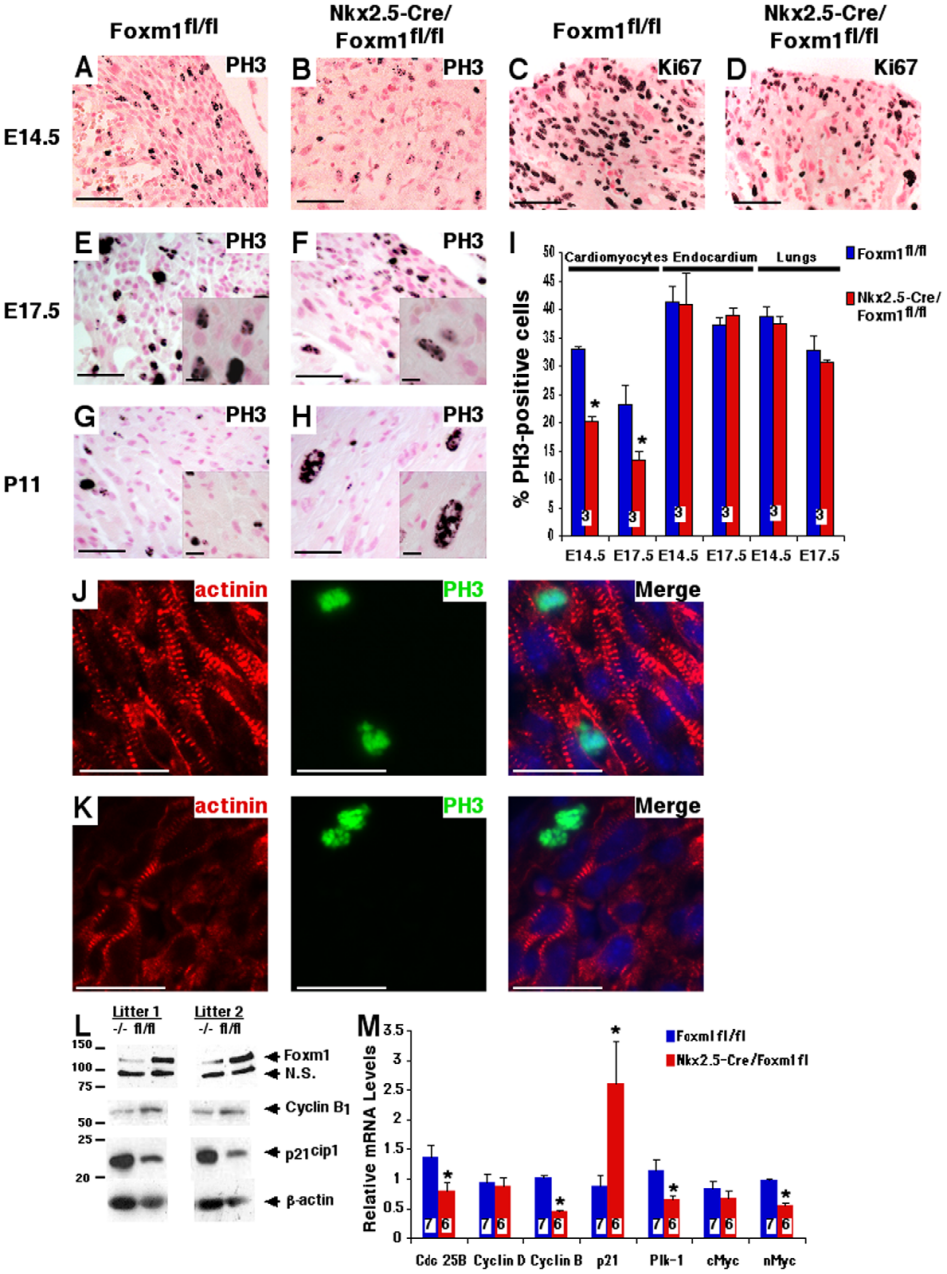
Decreased cardiomyocyte proliferation in *Nkx2.5-Cre/Foxm1^fl/fl^* hearts. Microtome sections of paraffin-embedded hearts were prepared from *Nkx2.5-Cre/Foxm1^fl/fl^* and control mice at E14.5 (A–D, J–K), E17.5 (E–F) and P11 (G–H) and stained for proliferation markers phospho-histone 3 (PH3) (A–B, E–H, J–K) and Ki-67 (C–D). The percent of PH3-positive cardiomyocytes was significantly lower in *Nkx2.5-Cre/Foxm1^fl/fl^* hearts compared to control at E14.5 and E17.5, though proliferation was unaltered in the lung and in endocardial cells (I). Proliferating cardiomyocytes were more prevalent in control (J) than in *Nkx2.5-Cre/Foxm1^fl/fl^* hearts (K) as evidenced by colocalization of PH3-positive nuclei with α-actinin. Consistent with decreased proliferation in *Nkx2.5-Cre/Foxm1^fl/fl^* hearts, there was decreased mRNA expression of the cell cycle regulators Cdc25B, Cyclin B_1_, Plk-1 and nMyc as determined by qRT-PCR (M). There was no change in the expression of Cyclin D_1_ or cMyc. Also consistent with decreased proliferation, there was increased mRNA expression of the cell cycle inhibitor p21^cip1^. Gene expression was determined using whole heart RNA and normalized to β-actin mRNA. Decreased gene expression of Foxm1, Cyclin B_1_, and increased p21^cip1^ expression translated to changes in protein levels as demonstrated by Western blot analysis (L). β-actin was used as a loading control. Significant differences (p<0.05) were indicated by asterisk. “N” values were represented by boxes inside bars. Scale bars represent 50 µm in A–H and J–K and 10 µm in insets E–H.

Cardiac proliferation was examined in *Nkx2.5-Cre/Foxm1^fl/fl^* and control embryos by immunohistochemical staining with antibodies against either phospho-histone 3 (PH3) or Ki-67. PH3-positive cardiomyocytes undergoing mitosis were observed in both *Nkx2.5-Cre/Foxm1^fl/fl^* and control *Foxm1^fl/fl^* mice at all timepoints studied (Figure 4A–H). However, cardiomyocyte proliferation was significantly diminished in *Nkx2.5-Cre/Foxm1^fl/fl^* hearts compared to littermate controls as indicated by significant decreases in the percentage of PH3-positive cardiomyocytes of 39% at E14.5 and 43% at E17.5 (Figure 4I). Yet, there was no change in cellular proliferation within the lung or in endocardial cells at these same timepoints (Figure 4I), indicating that proliferation defects were restricted to cardiomyocytes in *Nkx2.5-Cre/Foxm1^fl/fl^* embryos. Consistent with decreased numbers of PH3-positive cardiomyocytes, *Nkx2.5-Cre/Foxm1^fl/fl^* hearts displayed a dramatic reduction in Ki-67, a proliferation-specific protein (Figure 4C–D). Furthermore, decreased mRNA expression of cell cycle regulators Cdc25B, Cyclin B_1_, Polo-like kinase 1 (Plk-1) and nMyc was found in E14.5 *Nkx2.5-Cre/Foxm1^fl/fl^* hearts by qRT-PCR (Figure 4M). There was no change in the expression of Cyclin D_1_ or cMyc (Figure 4M). In addition, *Nkx2.5-Cre/Foxm1^fl/fl^* hearts displayed increased mRNA levels of p21^cip1^ (Figure 4M), a known cell cycle inhibitor critical for activity of cyclin-dependent kinase 2 (cdk2). Western blot analysis confirmed decreased protein levels of Foxm1 and Cyclin B_1_ as well as increased p21^cip1^ expression in *Nkx2.5-Cre/Foxm1^fl/fl^* hearts (Figure 4L). These results demonstrated that Foxm1 deletion from cardiomyocytes altered expression of cell cycle regulatory genes, contributing to proliferation defects and structural abnormalities in the developing heart.

### Decreased capillary density and cardiac fibrosis in postnatal *Nkx2.5-Cre/Foxm1^fl/fl^* hearts

**Figure 5:**
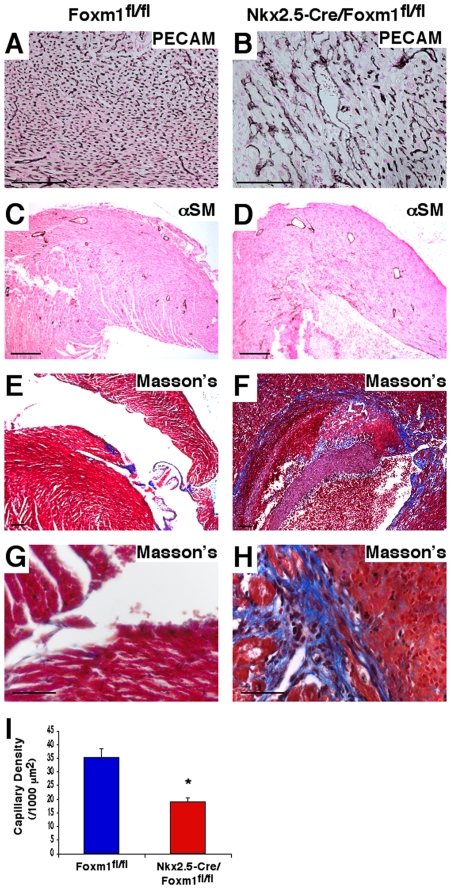
Decreased capillary density and cardiac fibrosis in *Nkx2.5-Cre/Foxm1^fl/fl^* heart. Microtome sections of paraffin-embedded hearts were prepared from either *Nkx2.5-Cre/Foxm1^fl/fl^* or littermate control mice at P11 and stained for PECAM-1 (A–B), α-smooth muscle actin (αSM) (C–D) or Masson's Trichrome (E–H). Capillary density was decreased in *Nkx2.5-Cre/Foxm1^fl/fl^* hearts at P11 (A–B, I), while coronary vessel formation was not altered (C–D). Numbers of capillaries were counted in PECAM-stained hearts using 10 random sections and mean±SEM was determined to confirm decreased capillary density (I). Significant fibrosis was detected in the IVS and ventricular walls of *Nkx2.5-Cre/Foxm1^fl/fl^* hearts (F, H) while none was detected in control hearts (E, G). Significant differences (p<0.05) were indicated by asterisk. Scale bars represent 100 µm in A–F and 50 µm in G–H.

Microtome sections of paraffin-embedded hearts from *Nkx2.5-Cre/Foxm1^fl/fl^* and control mice were subjected to immunohistochemical staining with antibodies against PECAM-1 or α-smooth muscle actin (αSM). PECAM-1 staining indicated a clear paucity in capillary density in the *Nkx2.5-Cre/Foxm1^fl/fl^* mouse heart at P11 (Figure 5A–B). Subsequent quantification showed a greater than 50% decrease in capillary density in the *Nkx2.5-Cre/Foxm1^fl/fl^* heart compared to *Foxm1^fl/fl^* control at P11 (Figure 5I). Despite the decreased capillary density, there was no difference in the number or morphology of coronary vessels in *Nkx2.5-Cre/Foxm1^fl/fl^* mouse hearts as indicated by αSM staining (Figure 5C–D). In addition, microtome sections from *Nkx2.5-Cre/Foxm1^fl/fl^* hearts were stained with Masson's Trichrome to detect cardiac fibrosis. Although there were no obvious signs of cardiac fibrosis at E17.5 (data not shown), the postnatal *Nkx2.5-Cre/Foxm1^fl/fl^* mouse heart displayed extensive fibrotic depositions in the interventricular septum as well as the apex of the ventricles (Figure 5F and H). No fibrosis was observed in postnatal *Foxm1^fl/fl^* control hearts (Figure 5E and G). Thus, Foxm1 deletion from cardiomyocytes altered vascular organization and induced cardiac fibrosis.

### Foxm1 deletion from cardiomyocytes caused decreased expression of CaMKIIδ, NFATc3, myocardin and Hey2

**Figure 6:**
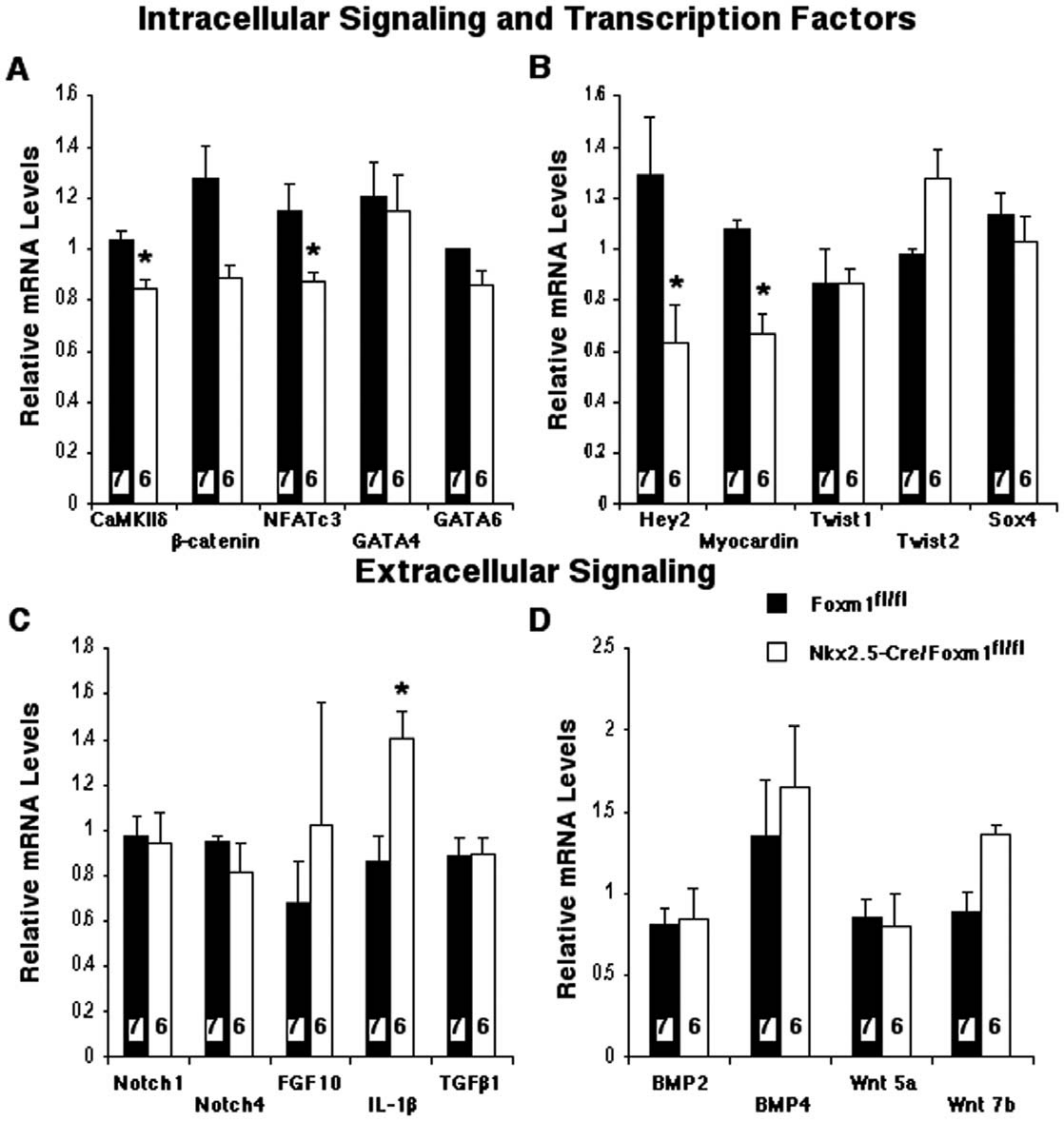
Reduced mRNA expression of CaMKIIδ, NFATc3, Hey2 and myocardin in *Nkx2.5-Cre/Foxm1^fl/fl^* hearts. Whole-heart RNA was isolated from embryonic *Nkx2.5-Cre/Foxm1^fl/fl^* and control *Foxm1^fl/fl^* hearts and used for qRT-PCR analysis. Taqman primers specific for CaMKIIδ, β-catenin, NFATc3, GATA4, GATA 6, Hey2, myocardin, Twist1, Twist2, Sox4, Notch1, Notch4, FGF10, IL-1β, TGF-β_1_, BMP2, BMP4, Wnt5a and Wnt7b (Table 1) were used to evaluate cardiac mRNA expression. Gene expression was normalized to β-actin mRNA. Significantly diminished mRNA levels of the intracellular signaling molecule CaMKIIδ and transcription factors NFATc3, Hey2 and myocardin were detected in *Nkx2.5-Cre/Foxm1^fl/fl^* hearts. Increased mRNA expression of IL-1β was also detected. Significant differences (p<0.05) were indicated by asterisk. “N” values were represented by boxes inside bars.

To determine new potential Foxm1 target genes in the developing heart, expression of transcription factors and signaling molecules critical for heart morphogenesis or Foxm1 signaling was examined by qRT-PCR. *Nkx2.5-Cre/Foxm1^fl/fl^* embryos displayed a significant decrease in cardiac expression of NFATc3 (Figure 6A), a known Foxm1 target gene [13]. These results are consistent with reduced expression of NFATc3 in Foxm1-depleted ML-1 cells and hearts from *Foxm1^−/−^* mice [13]. Embryonic *Nkx2.5-Cre/Foxm1^fl/fl^* hearts exhibited normal mRNA expression of the intracellular signaling molecule β-catenin and the transcription factors GATA4, GATA6, Twist1, Twist2 and Sox4 (Figure 6A–B). Although interleukin-1β mRNA was increased in *Nkx2.5-Cre/Foxm1^fl/fl^* hearts (Figure 6C), there was no change in mRNA levels of other extracellular signaling molecules critical for heart morphogenesis, such as Notch1, Notch4, FGF10, TGF-β_1_, BMP2, BMP4, Wnt 5a or Wnt 7b (Figure 6C–D).

Expression of calcium/calmodulin-dependent kinase IIδ (CaMKIIδ) was significantly reduced in *Nkx2.5-Cre/Foxm1^fl/fl^* hearts (Figure 6A). CaMKIIδ is an intracellular signaling molecule that is known to play a critical role in beat-to-beat cardiac physiology and has been shown to be an essential mediator of pressure overload and pharmacologically induced hypertrophy [37]. In addition, CaMKIIδ mediates calcium signaling and growth mechanisms within the cardiomyocyte [38]. Our results suggest that CaMKIIδ is a downstream effector of Foxm1 signaling as well as a contributing factor in myocardial thinning and embryonic lethality associated with cardiomyocyte-specific Foxm1 deletion.

Expression of both Hey2 and myocardin was reduced in *Nkx2.5-Cre/Foxm1^fl/fl^* hearts (Figure 6B). Hey2 knockout mice exhibited a thin walled left ventricle and decreased cardiomyocyte proliferation [8], similar to the phenotype in *Nkx2.5-Cre/Foxm1^fl/fl^* mice. Myocardin is a cofactor for the serum response factor (SRF) and is essential for cardiogenesis [39], [40]. Furthermore, SRF knockout mice exhibited a thin myocardium with dilated chambers and disorganized IVS [41], a phenotype similar to *Nkx2.5-Cre/Foxm1^fl/fl^* hearts. Therefore, decreased expression of Hey2 and myocardin may contribute to the cardiac abnormalities and embryonic lethality of the *Nkx2.5-Cre/Foxm1^fl/fl^* mouse. Altogether, our results demonstrate that Foxm1 deletion from cardiomyocytes alters expression of cardiac genes critical for heart morphogenesis and cardiomyocyte proliferation.

## Discussion

We have previously generated and characterized a Foxm1-null (*Foxm1^−/−^*) mouse line in which mice die between embryonic days E13.5 and E16.5 due to severe defects in multiple organ systems including lungs, liver, heart and blood vessels [25]. We further demonstrated that tissue-specific deletion of Foxm1 from hepatocytes [25], respiratory epithelium [12] or smooth muscle cells [28] was sufficient to cause lethality *in utero* or shortly after birth. Therefore, Foxm1 is essential for organ morphogenesis in multiple organ systems. However, it remained to be determined whether cardiac malformation in *Foxm1^−/−^* embryos was due to cardiomyocyte derived effects of Foxm1 signaling or if these Foxm1^−/−^ defects were indirect, resulting from abnormalities in other organ systems and altered embryonic homeostasis. To elucidate the cardiomyocyte-autonomous role of Foxm1 in embryonic heart development, we used the Cre-LoxP system to generate a conditional Foxm1 knockout mouse line in which Foxm1 is selectively deleted from cardiomyocytes under control of the Nkx2.5 promoter, one of the earliest known cardiac markers.

Deletion of Foxm1 from cardiomyocytes caused thinning and disorganization of the muscular walls of the heart, including both ventricles and the interventricular septum. Myocardial thinning was due to decreased cardiomyocyte proliferation accompanied by altered expression of multiple cell cycle regulatory genes. Ultimately, the myocardial hypoplasia in *Nkx2.5-Cre/Foxm1^fl/fl^* embryos caused lethality in late gestation. Although the myocardial phenotype exhibited similarities to that observed in hearts from *Foxm1^−/−^* mice, many unique features were observed in the conditional knockout model suggesting cardiomyocyte-autonomous roles for Foxm1 signaling. These include a delay in the onset of lethality, unaltered heart size, diminished cardiac capillary density and myocardial fibrosis. The decrease in cardiomyocyte proliferation in *Nkx2.5-Cre/Foxm1^fl/fl^* embryos was significantly less than in *Foxm1^−/−^* embryos suggesting that abnormalities in other cell types or tissues contributed to cardiac malformation in mice with complete deletion of Foxm1. Although we observed increased deposition of extracellular matrix in the atrio-ventricular valves of *Nkx2.5-Cre/Foxm1^fl/fl^* hearts the size of the valves was unaltered. These results are in contrast to valve thickening in *Foxm1^−/−^* mice [13] and suggest that although Foxm1 does mediate atrio-ventricular valve formation, this signaling is not cardiomyocyte-dependent. Alternatively, valve defects *Foxm1^−/−^* mice may result from altered blood pressure caused by structural abnormalities in blood vessels that were previously reported [26].

To date, embryonic lethality associated with ventricular hypoplasia and myocardial thinning has been linked to several signaling cascades including the transcription factor Hey2 [8], members of the NFAT family [42] or inactivation of serum response factor (SRF) [41]. In this study we described a model of myocardial thinning owing to multiple factors and resulting in embryonic lethality. In addition to altered expression of various cell cycle regulatory genes, this study identified Hey2, myocardin and CaMKIIδ as novel targets of Foxm1 signaling *in vivo* and as potential mediators of the thin ventricular phenotype.

We previously showed decreased expression of NFATc3 in *Foxm1^−/−^* hearts and in Foxm1-depleted cardiomyocytes *in vitro* [13]. This study confirmed that Foxm1 is a positive regulator of cardiac NFATc3 expression and further identified cardiomyocytes as the cell type responsible for Foxm1-regulated NFATc3 expression *in vivo*. It has been previously shown that dual deletion of NFATc3 and NFATc4 causes thin ventricles, decreased proliferation of ventricular myocytes and pericardial effusion culminating in embryonic lethality [42]. Therefore, decreased NFATc3 expression could be a contributing factor in myocardial thinning and embryonic lethality associated with *Nkx2.5-Cre/Foxm1^fl/fl^* mice.

Decreased expression of Hey2 can contribute to the cardiac phenotype and embryonic lethality of *Nkx2.5-Cre/Foxm1^fl/fl^* mice as evidenced by embryonic lethality and myocardial thinning in Hey2^−/−^ mice, a phenotype similar to *Nkx2.5-Cre/Foxm1^fl/fl^* mice. Hey2 has also been shown to interact with the serum response factor (SRF) to inhibit activity of myocardin [43], which is essential for cardiogenesis, cardiomyocyte proliferation, migration and deposition of the extracellular matrix [39], [44], [45]. Furthermore, deletion of SRF from cardiomyocytes resulted in lethality between E10.5–13.5 with thin myocardium, dilated chambers and disorganized IVS [41], a phenotype similar to that observed in the *Nkx2.5-Cre/Foxm1^fl/fl^* hearts. Therefore, Foxm1 may directly influence myocardial development by decreasing expression of Hey2 and myocardin and possibly interfering with SRF-mediated signaling in cardiomyocytes.

CaMKIIδ deficiency caused augmented cardiac function in multiple heart injury models which manifested as severe alterations in cardiac structure. The finding of decreased CaMKIIδ mRNA in *Nkx2.5-Cre/Foxm1^fl/fl^* hearts suggests a role for Foxm1 in regulating cardiac CaMKIIδ expression. Interestingly, interleukin-1β (IL-1β) mRNA was increased in *Nkx2.5-Cre/Foxm1^fl/fl^* hearts. IL-1β has been reported to be a critical mediator of cardiac fibrosis [46]. Since significant fibrosis was observed in the postnatal *Nkx2.5-Cre/Foxm1^fl/fl^* heart, increased expression of IL-1β may contribute to fibrotic deposition.

In summary, we demonstrated that Foxm1 plays a cell-autonomous role in cardiomyocytes during cardiac development. Foxm1 deletion in developing cardiomyocytes caused embryonic lethality, decreased cardiomyocyte proliferation, diminished vascular density in the myocardium and induced cardiac fibrosis in the early postnatal period. This study further identified Hey2, myocardin, NFATc3, CaMKIIδ and various cell cycle regulatory genes as *in vivo* targets of Foxm1 signaling and potential mediators of the myocardial thinning and ventricular hypoplasia associated with the *Nkx2.5-Cre/Foxm1^fl/fl^* phenotype.
